# Circulating microRNA-144-5p is associated with depressive disorders

**DOI:** 10.1186/s13148-015-0099-8

**Published:** 2015-07-22

**Authors:** Xiao Wang, Kristina Sundquist, Anna Hedelius, Karolina Palmér, Ashfaque A. Memon, Jan Sundquist

**Affiliations:** Center for Primary Health Care Research, Lund University/Region Skåne, Malmö, Sweden; Stanford Prevention Research Center, Stanford University School of Medicine, Stanford, CA USA

**Keywords:** miR-144-5p, Depression, Severity

## Abstract

**Background:**

Depressive/anxiety disorders are the most common types of mental illnesses in the world. The present study was the first to explore the association between plasma microRNAs (miRNAs) and depression/anxiety in primary care patients.

**Results:**

In total, 169 patients (aged 20–64 years) from 16 primary health centers were enrolled in the present study. The healthy controls were consisted of 52 individuals. We first performed miRNA screening of plasma samples from 11 patients using a Serum/Plasma Focus microRNA Panel comprising 179 miRNA primer sets. Six miRNAs were differentially expressed and were then validated by quantitative real-time (qRT)-PCR in the entire study cohort. The mean plasma miR-144-5p level in the depression/anxiety patients increased significantly compared to baseline (*p* < 0.0001) after the 8-week follow-up. No significant associations were found between the differentially expressed miRNAs and a change in the Montgomery-Åsberg Depression Rating Scale (MADRS-S) score after the follow-up. In linear regression analysis, the plasma miR-144-5p expression level was inversely related to the depression score (MADRS-S) (*β* = −0.02, *p* < 0.01), after adjustment for sex and age, at baseline. In addition, plasma miR-144-5p levels at baseline in the depression/anxiety patients were significantly lower compared with the healthy controls (*p* < 0.001).

**Conclusions:**

Our findings show that plasma miR-144-5p levels are associated with depressive symptoms. Although confirmatory analyses are required, plasma miRNA-144-5p is a potential peripheral biomarker for pathologic processes related to depression.

**Electronic supplementary material:**

The online version of this article (doi:10.1186/s13148-015-0099-8) contains supplementary material, which is available to authorized users.

## Background

Depression and anxiety are common mental disorders. The World Health Organization (WHO) has predicted that depression will become the second leading contributor to disease burden by the year 2020 [1]. In different European studies, approximately 15 to 32 % of patients who visited primary health care centers showed symptoms of depression, anxiety, and stress-related disorders [2–5]. These in primary health care common psychiatric disorders are associated with poor quality of life and reduced life expectancy, placing a large economic burden on society. Several studies have explored the underlying pathophysiological and etiological mechanisms [6–8]. Current evidence suggests that the onset and progression of depression appear to be influenced by multiple factors, including neurotransmitter systems [9, 10], stress [7, 11], and genetic susceptibility [7, 12]. However, the molecular pathophysiology underlying depression and anxiety is still not fully understood. In recent years, the emergence of small noncoding RNAs as regulators of gene expression have gained much attention in various disease pathophysiologies. MicroRNAs are some of the most studied and well characterized among small noncoding RNAs. [13–17].

MicroRNAs (miRNAs) are a class of small (21-23-nucleotide), noncoding, single-stranded RNAs that inhibit gene expression by promoting messenger-RNA (mRNA) degradation or inhibiting translation [18]. They influence a variety of physiological cell processes during development and tissue homeostasis by regulating the expression of around 90 % of all human genes [19]. MicroRNAs are highly expressed in neurons, where they regulate processes of brain development, including neurogenesis, neuronal proliferation, metabolism, and apoptosis [20]. Aberrant expression or dysregulation of miRNA processing has been linked to many neurological and psychiatric diseases. Studies in postmortem brains from patients with Alzheimer’s disease, schizophrenia, bipolar disorder, and major depressive disorders showed alterations in several miRNAs [21–24]. Recently, numerous miRNAs have been detected in several body fluids, including serum, plasma, and cerebrospinal fluid (CSF) [25]. The profile of circulating miRNAs changes significantly under pathological conditions compared to healthy conditions. Circulating miRNAs can be secreted from cells into the blood in different ways—enclosed in exosomes or associated with proteins [26–29]. They are resistant to nuclease digestion, and can be measured reproducibly, which makes them attractive as potential biomarkers.

Over the past several years, circulating miRNAs, as potential biomarkers, have been well documented in many diseases, including cancer, diabetes, and psychiatric diseases [13–15, 30–32]. Recently, two studies have examined circulating miRNA expression in the blood in patients with major depressive disorder [31, 33] and found some dysregulated miRNAs. However, these studies only had small numbers of patients with a focus on major depressive disorders. A systematic analysis of plasma miRNAs in depression and anxiety has so far not been performed.

The present study builds on a previously published randomized controlled trial by our group, where we included patients with depression/anxiety from 16 primary health care centers [34] in Sweden in order to compare the effects of mindfulness treatment with treatment as usual on depressive/anxiety symptoms. In the present study, we hypothesized that patients with depression/anxiety would experience a significant change in plasma miRNAs after an 8-week treatment and that there would be an association between plasma miRNA levels and symptoms of depression/anxiety at baseline. Using a broad miRNA screening panel, we identified a group of miRNAs whose expression differed between baseline and follow-up using plasma samples from 11 patients who showed a decline in the Montgomery-Åsberg Depression Rating Scale (MADRS-S) total score of at least 50 % between baseline and follow-up. The first aim was to examine whether the miRNAs that were identified as differentially expressed after treatment were associated with a response to treatment. The second aim was to examine the association between these differentially expressed miRNAs and symptoms of depression/anxiety at baseline.

## Results

### Patients’ characteristics

The clinical characteristics of the patients are shown in Table 1. The mean age in the whole group was 42 years and more of the participants were women. Most antidepressants prescribed at primary health care centers in Sweden are selective serotonin reuptake inhibitor (SSRIs) (e.g., citalopram, fluoxetine, and sertraline, data not shown). The median scores at baseline indicated mild to moderate symptoms of depression and/or anxiety. After treatment, the median scores decreased, indicating none to mild symptoms. A total of 39 % of the patients had a decrease in MADRS-S score of at least 50 % after the 8-week treatment compared to baseline (data not shown in tables).

**Table 1:** Characteristics of the patients in the whole cohort

Variable	The whole cohort (*n* = 169)
Baseline MADRS-S	20 (11)
Median score (IQR)	
Follow-up MADRS-S	11 (10)
Median score (IQR)	
Baseline HADS-D	8 (5)
Median score (IQR)	
Follow-up HADS-D	4 (4)
Median score (IQR)	
Baseline HADS-A	12 (5.7)
Median score (IQR)	
Follow-up HADS-A	7 (4)
Median score (IQR)	
Baseline PHQ-9	12 (9)
Median score (IQR)	
Follow-up PHQ-9	6 (6)
Median score (IQR)	
Age, years	42 (11)
Mean (SD)	
Sex	13/86
Male/female (%)	
Antidepressants^a^	34/57
Yes/no (%)	
Tranquilizers^a^	14/72
Yes/no (%)	

*IQR* interquartile range, *SD* standard deviation

^a^16 patients had missing data for antidepressants, and 24 had missing data for tranquilizers

### miRNAs screening data

To increase the chances of identifying miRNAs that are associated with treatment response, we performed initial screening of plasma from 11 patients with at least a 50 % decline in MADRS-S total score at follow-up. Six candidate miRNAs with a >1.5 fold differential expressions between baseline and follow-up were selected (Additional file 1: Table S1): miR-144-5p, miR-92b-3p, miR-885-5p, miR-30a-5p, miR-29a-5p, and miR-29b-2-5p. These six miRNAs were then measured in the entire cohort. Five of them were detectable in all samples, but miR-885 was not suitable for further analysis as more than 20 % of patients had Ct values of >35.

### Response analysis

The selected five miRNAs were validated in all of the 169 patients at baseline and follow-up. Additional file 2: Figure S1 shows that after treatment, the mean plasma miR-144-5p level and miR-30a-5p in the depression/anxiety patients increased significantly compared to baseline, (*p* < 0.0001 and *p* = 0.007, respectively). Moreover, to assess the potential association between candidates’ miRNAs and treatment response, we performed linear regression analysis between miRNAs changes (∆∆Ct) and MADRS score change in the patients. However, no significant associations were found between the changes of those selected miRNAs and MADRS-S score change after treatment (Table 2).

**Table 2:** Associations between selected miRNAs (∆∆Ct = ∆Ct_follow-up_-∆Ct_baseline_) and change in MADRS-S for all patients, adjusted for MADRS-S at baseline, as determined using linear regression

	Univariate analysis		
	*β*	*p* value	95 % CI
miR-144-5p (*n* = 160)			
MADRS-S	−0.004	0.68	−0.02; 0.01
miR-29a-5p (*n* = 153)^a^			
MADRS-S	−0.005	0.65	−0.02; 0.02
miR-*29b*-*2*-*5p* (*n* = 153)^a^			
MADRS-S	−0.002	0.82	−0.02; 0.02
miR-30a-5p (*n* = 153)^a^			
MADRS-S	0.003	0.72	−0.01; 0.02
miR-92b-3p (*n* = 160)^a^			
MADRS-S	−0.005	0.67	−0.03; 0.02

^a^Information (clinical or qRT-PCR) is missing for some patients (Tables 2 and 3)

### Associations of miRNAs with symptoms of depression/anxiety (cross-sectional study)

Unadjusted linear regression revealed that plasma miR-144-5p expression level at baseline was inversely related to MADRS-S score at baseline (*β* = −0.02, *p* < 0.01) (Table 3). Adjustment for sex and age did not affect these values. The lower the plasma miR-144-5p expression levels were the higher was the depression score. Like miR-144-5p, miR-29a-5p showed a significant positive association with MADRS-S score (*β* = 0.02, *p* = 0.04) in the unadjusted regression. After adjustment for age and sex, the *p* value (0.06) no longer remained significant (Table 3). We also corrected for multiple testing according to Bonferroni. We adjusted the individual *p* value for each of these five miRNAs and found that the association between miR-144-5p and the MADRS-S at baseline was still significant. The relationships between miR-144-5p and the scores at baseline are also shown for the other two scales (Additional file 1: Table S2a, b). The overall pattern for the other two scales was similar to the results for MADRS-S.

**Table 3:** Associations between selected miRNAs (∆Ct at baseline) and MADRS-S for the whole cohort, as determined using linear regression

	Univariate analysis			Adjusted analysis		
	*β*	*p* value	95 % CI	*β*	*p* value	95 % CI
miR-144-5p (*n* = 166)						
MADRS-S at baseline	−0.02	0.009	−0.03; −0.005	−0.02	0.008	−0.03; −0.005
Sex (female vs. male)				−0.34	0.03	−0.64; −0.04
Age				0.005	0.28	−0.004; 0.01
miR-29a-5p (*n* = 159)^a^						
MADRS-S at baseline	0.02	0.04	0.0004; 0.03	0.01	0.06	−0.0005; 0.03
Sex (female vs. male)				−0.007	0.97	−0.35; 0.34
Age				−0.01	0.04	−0.02; −0.0007
miR-29b-2-5p (*n* = 159)^a^						
MADRS-S at baseline	0.004	0.62	−0.01; 0.02	0.004	0.61	−0.01; 0.02
Sex (female vs. male)				−0.18	0.30	−0.53; 0.16
Age				0.005	0.39	−0.006; 0.01
miR-30a-5p (*n* = 159)^a^						
MADRS-S at baseline	0.002	0.73	−0.01; 0.02	0.003	0.68	−0.01; 0.02
Sex (female vs. male)				0.24	0.14	−0.07; 0.55
Age				0.0006	0.90	−0.009; 0.01
miR-92b-3p (*n* = 166)^a^						
MADRS-S at baseline	0.01	0.11	−0.002; 0.02	0.01	0.12	−0.002; 0.02
Sex (female vs. male)				0.04	0.78	−0.23; 0.30
Age				−0.003	0.51	−0.01; 0.005

^a^Information (clinical or qRT-PCR) is missing for some patients (Tables 2 and 3)

### Comparison of miR-144-5p expression in depression/anxiety patients and healthy controls

**Fig. 1:**
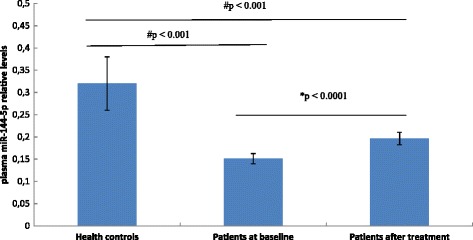
Plasma miR-144-5p levels (determined by the 2ΔCt method): data are shown as the mean and 95 % CI. *p: paired *t* test), #*p*: linear regression

Figure 1 shows the relative plasma miR-144-5p expression levels in the healthy controls and the depression/anxiety patients before and after the 8-week treatment. The relative miR-144-5p expression levels at both baseline and after treatment were significantly lower in the depression/anxiety patients than in the healthy controls (*p* < 0.001). In the depression/anxiety patients, the mean plasma miR-144-5p level increased significantly after treatment compared to baseline (*p* < 0.0001).

## Discussion

To our knowledge, this is the first study to investigate the expression of miRNAs in primary health care patients with depression/anxiety. Our major findings are that plasma expression levels of miR-144-5p were inversely associated with depression scores at baseline and that plasma miR-144-5p levels in depressive patients were significantly lower than in healthy controls. These findings suggest that miRNA-144-5p may reflect the pathologic processes of depression. Moreover, we found that plasma levels of miRNA-144-5p were significantly higher after treatment compared to baseline levels in depression/anxiety patients. No significant associations were found between the changes of those differentially expressed miRNAs and MADRS-S score change after treatment (response analysis).

The finding of an inverse correlation between plasma miR-144-5p levels and the MADRS-S at baseline was also present for the other two depression scores, i.e., Hospital Anxiety and Depression Scale (HADS)-D and PHQ-9; however, the results for HADS-D and PHQ-9 did not reach statistical significance. We applied correction for multiple testing according to Bonferroni, and the lack of significance for HADS-D and PHQ-9 may be due to the sample size in combination with the Bonferroni correction. An advantage with the MADRS-S is that it is a commonly used scale for depression [35, 36], which is sensitive to discriminate the severity of depression and it is therefore preferred in studies where the intention is to measure core depressive symptoms and states [37].

The presentation of depressive syndromes is heterogeneous. Thus, identifying patients with depression using only depression scores or patient self-report may lead to a delayed or even wrong diagnosis [38, 39]. It is thus necessary to identify stable biomarkers to help with the clinical diagnosis of depression and elucidate potential biological mechanisms. A stable biomarker that is easily assessed in peripheral blood might be an ideal and objective measure to diagnose or evaluate the stage of depression [40]. Such biomarkers are still lacking. Circulating miRNAs are stable and easily accessible, which may enable their application in daily clinical practice. Studies have reported that circulating miRNAs are released from specific cells and transferred to recipient cells to exert their function [41]. Furthermore, aberrant expression of circulating miRNAs was demonstrated to be related to tissue injury [42], which raises the possibility of blood-based miRNA profiles as fingerprints of diseases. Based on the evidence described above, the notion of circulating miRNAs as biomarkers for various diseases has been expressed.

Strengths of our study are that the present results were obtained from an randomized controlled trial (RCT) including a total of 169 patients, who are representative of patients in primary health care. Furthermore, this is the first study to provide evidence that a specific miRNA may serve as a potential biomarker for the diagnosis of depression. Presently, the role of circulating miR-144-5p in depressive disorders remains unclear. However, there are a few biological mechanisms that can explain our finding.

MiR-144 has broad expression and is enriched in brain, as well as in normal and malignant hematopoietic cells and tissues [43]. It is highly conserved and has multiple predicted targets in both humans and rats [44]. Many studies have demonstrated that miR-144 is involved in the response to mood stabilizer treatment [45], stress responses [46, 47], and aging diseases [48]. Long-term treatment with the mood stabilizers lithium and valproate significantly up-regulates miR-144 expression in the rat hippocampus, which is one of the brain regions involved in mood regulation. Signaling pathways targeted by miR-144 include the protein kinase C (PKC), Wnt/β-catenin, and PTEN pathways [45]. Some of them have been shown to be involved in the development of depression [49, 50]. In addition, miR-144 was reported to be selectively up-regulated in the aging brain of humans and was suggested to have neuroprotective functions. miR-144 can inhibit the expression of ataxin 1 (ATXN1) in human cells, and a search of the Genetic Association Database shows that ATXN1 is associated with mental disorders, such as bipolar disorder, schizophrenia, and major depressive disorder [48]. It is known that stress is an important risk factor for depression. In one study, circulating miR-144* level changed with stress associated with the nationally administered examination for academic promotion in healthy young adults: miR-144* level was significantly increased after the examination compared to before the examination [46]. However, there was no correlation between miR-144* level and anxiety scores [46], which is consistent with our findings (Additional file 1: Table S2b). We assume that the increased circulating miR-144 level may reflect changes in miR-144 expression in the brain, which may in turn be associated with the progression of depression. The psychopathological mechanisms of the effect of miR-144 on depression remain to be elucidated. Our results, however, is a complement to the recent findings that decreased levels of miR-144* are associated with several diseases [51, 52].

Our study is the first RCT to explore the association of circulating miRNA expression in depression. Despite our promising results, there are several general limitations to our experimental design that should be considered when interpreting our results. First, we included patients with depression as well as anxiety. Overlapping symptoms could affect our findings. However, overlapping symptoms are relatively common among these conditions and our approach is in line with previous studies. Second, in the initial screening analysis, we ran one well instead of duplicates/triplicates for each sample. However, when we setup technical replicates (duplicates or triplicates) in our laboratory, the technical variation was very small. Another limitation is that only six miRNAs were selected from the initial miRNA screen and validated in the whole cohort, suggesting that other miRNAs that are potentially associated with depression scores could have been missed in the current study. However, these selected miRNAs were the only miRNAs that were differentially expressed. In addition, this is the first study to examine miRNAs’ potential association with depression/anxiety, and there is so far no available standard for selection of candidate miRNAs for screening. In addition, the correlation between plasma miR-144-5p expression level and miR-144-5p in the brain is unknown, as is the source of plasma miR-144-5p.

## Conclusions

In summary, our findings show that plasma miR-144-5p levels are associated with depressive symptoms in a primary health care setting. In further studies, plasma miR-144-5p levels need to be measured in patients in other clinical settings, including major depressive disorders, to examine the validity of our findings. It will also be of interest to examine whether the expression of exosomal miR-144-5p is related to depression scores. In addition, it would be informative to test the predictive value of miR-144-5p in a larger cohort of patients with depression.

## Methods

### Study subjects and sample collection

The study subjects were recruited from 16 primary health care centers in a RCT of mindfulness group therapy compared to treatment as usual. The RCT included a group of patients with depression, anxiety or stress, and adjustment disorder. A detailed description of the study design is provided in our previous article [34]. The overall aim of the RCT was to compare the effect of a structured mindfulness-based group therapy program with treatment as usual (mostly individual cognitive behavioral therapy (CBT)) [53]. Briefly, patients were recruited between 4 Jan 2012 and 22 March 2012 at the 16 primary health centers in urban and rural settings in Skåne, in the southernmost part of Sweden. The inclusion criteria were as follows: age 20–64 years and a score of ≥10 on the Patient Health Questionnaire (PHQ)-9 or ≥7 on the HADS or a total score on the MADRS-S of between 13 and 34 (mild to moderate depression). A total of 135 patients met the inclusion criteria for MADRS-S, 118 for HADS-A, 159 for HADS-D, and 111 for PHQ-9. The rationale for using multiple scales to assess symptoms of depression and anxiety was that different scales are used in clinical practices worldwide [34]. Eligible patients had a clinical diagnosis of depression, anxiety or stress, and adjustment disorder, according to the International Classification of Diseases (ICD)-10 criteria. All clinical diagnoses were made by doctors at the 16 primary health care centers. The exclusion criteria were as follows: severe personality disorder, risk of suicide, pregnancy, thyroid disease, current psychotherapy of any kind, and participation in any other psychiatric intervention study. The clinical characteristics and miRNA distributions of the total study population are presented in Table 1. In total, 169 patients (86 % females, 13 % males, 1 % gender not specified) aged 42 ± 11 years (mean ± standard deviation) were enrolled in the present study. All the patients were fluent in Swedish, and 90 % were born in Sweden and/or had at least a high school degree (not shown in table). Each patient filled in three self-rated questionnaires (above mentioned PHQ-9, HADS-A/HADS-D, and MADRS-S) at baseline and after 8 weeks of follow-up. The patients received antidepressants and tranquilizers if deemed necessary. Blood samples were collected at the same time as the assessment of self-rated symptoms before and after treatment. The control group was consisted of 52 healthy individuals (74 % females, 26 % males) aged 47 ± 12 years. They were recruited from all types of personnel groups among the hospital staff.

### Plasma collection

For patients, whole blood (6 mL) was collected from each participant in EDTA tubes. Blood samples were centrifuged at 2000 g for 10 min at 4 °C, and the plasma was then aliquoted and stored at −80 °C before further processing. For healthy controls, whole blood was collected in buffered citrate tubes and centrifuged at 2000 g for 20 min at room temperature. Blood samples were processed and plasma frozen within 8 h of collection.

### Sampling strategy and miRNA analysis

For the miRNA screening, 11 individuals (8 females and 3 males, age 41 ± 9. 2 years) with a minimum reduction in MADRS-S score of 50 % from the initial evaluation were selected. We selected those individuals who showed the largest change in the magnitude of the depressive symptoms. We assumed that a linear change in the magnitude of the depressive symptoms would also be associated with the magnitude of the depressive/anxiety symptoms at baseline.

We used MADRS-S score as our selection criteria because it is a commonly used scale for depression [31, 35, 36, 54]. The individuals in this initial screening analysis were chosen to resemble the whole group as much as possible, based on sex and age. Fifty microliter of total RNA was isolated from 200 μl of plasma using the Qiagen miRNeasy Mini Kit (Qiagen GmbH, Hilden, Germany) according to the manufacturer’s protocol, with minor modifications. miRNAs were reverse transcribed using a Universal cDNA Synthesis kit (Exiqon, Vedbæk, Denmark). The resulting reverse transcription reaction product was stored at −20 °C before analysis. A detailed description of the methodology is provided in a previous article [30]. miRNA expression was screened using a Serum/Plasma Focus microRNA PCR Panel (Exiqon) comprising 179 LNA™ microRNA primer sets focusing on serum/plasma-relevant human miRNAs. Quantitative real-time PCR (qPCR) was conducted using a CFX384 Real-Time PCR Detection System (Bio-Rad). Undetectable data were assigned a default threshold cycle (Ct) value of 36. A mean of 172 miRNAs were detected in the 11 samples. As there is no current consensus as to an appropriate reference miRNA for the normalization of plasma miRNAs in qPCR analysis, all qPCR data were normalized to the Ct average of all miRNA measurements for each sample [55]. Comparison of the 172 miRNAs in the 11 samples revealed six miRNAs with >1.5 fold differential expression between baseline and follow-up (Additional file 1: Table S1). We used a >1.5 fold change between baseline and follow-up as the cutoff, which is based on several previous profiling studies confirming that a >1.5 fold change in miRNA expression can have a significant impact on the biology of the cell [55–57]. These six miRNAs were then measured in duplicate by qPCR in the whole cohort (*n* = 169). miR-451a and miR-23a-3p were used to test hemolysis in plasma samples [58]. The Ct values were normalized according to the ∆Ct method with the internal controls miR-425-5p and miR-186-5p. The normalization stability of those two miRNAs was confirmed with geNorm software [59]. Ct values were normalized to miR-425-5p and miR-186-5p using the following equation: ∆Ct = Ct_miR-425-5p_&_miR-186-5p_ (geometric mean of two miRNAs) − Ct_miR of interest_. Relative expression was calculated as 2^∆Ct^.

### Ethical considerations

The study was performed according to the principles of the Declaration of Helsinki. It was reviewed and approved by the Ethics Committee of Lund University, prior to its commencement, on 5 October 2011 (application no. 2011/491). Written informed consent was obtained from all participants.

### Statistical analysis

For the miRNA screening analysis, we tested the difference between baseline and follow-up using the Wilcoxon signed-rank test due to the nature of data, repeated measurements and nonnormality (Additional file 1: Table S1). MADRS-S, HADS-D (depression), HADS-A (anxiety), and PHQ-9 scores are presented as the median and interquartile range (IQR); age is presented as the mean and standard deviation (SD); and sex and antidepressant and tranquilizer use are presented as numbers and percentages (Table 1). The mean and 95 % confidence interval (CI) at baseline and follow-up are shown for plasma miR-144-5p levels. A paired *t* test was used to test the difference between baseline and follow-up (Additional file 2: Figure S1). The association between change in miRNA levels and treatment response was tested using linear regression, adjusted for MADRS-S at baseline (Table 2). Linear regression was also used to examine the associations between miRNA levels at baseline and MADRS-S, HADS-D, HADS-A, and PHQ-9 scores (Table 3 and Additional file 1: Table S2a-S2c). We considered important potential confounders to be sex, age, drug treatment (antidepressant and/or tranquilizer), body mass index (BMI), and smoking. When adjusting the models for these variables, the beta-coefficients did not change much (at the most 5 %) and no *p* values were significant for drug treatment, BMI, or smoking. Hence, we kept in the final models only sex and age as adjusting variables.

Linear regression analyses (adjusted for age and sex) were used to test the difference of miR-144-5p levels between healthy controls and patients at baseline and after treatment (Figure 1). STATA version 12 (StataCorp LP) was used for all statistical analyses.

## Additional files

Additional file 1:
**Supplementary Tables. Table S1.** six candidate miRNAs with >1.5 fold differential expression between baseline and follow-up and changes in more than half of the samples (n≥6) were selected for further validation. **Table S2a and Table S2b.** The relationships between miR-144-5p and two depression scores at baseline, HADS-D (β=-0.02, p=0.02) and PHQ-9 (β=-0.02, p=0.06) were also significant or borderline significant, whereas the association with HADS-A score (β=-0.01, p=0.37) was non-significant **(Table S2c)**. Additional file 2: Figure S1. Supplementary Figure. Plasma 5 miRNAs levels (determined by the 2ΔCt method)changed after treatment. Data are shown as the mean and 95 % CI. P calculated with paired t-test

## Abbreviations

ATXN1: ataxin 1
BMI: body mass index
CBT: cognitive behavioral therapy
CI: confidence interval
HADS: Hospital Anxiety and Depression Scale
ICD-10: International Classification of Diseases 10
IQR: interquartile range
MADRS-S: Montgomery-Åsberg Depression Rating Scale
miRNAs: microRNAs
PHQ-9: Patient Health Questionnaire
PKC: protein kinase C
qPCR: quantitative real-time
RCT: randomized controlled trial
SD: standard deviation
SSRIs: selective serotonin reuptake inhibitor

## References

[1] WHO (2001) Mental health, A call for action by World Health Ministers. Ministerial Round Tables 2001 5 4th WORLD HEALTH ASSEMBLY.

[2] Vazquez-Barquero JL, Garcia J, Simon JA, Iglesias C, Montejo J, Herrán A, et al. Mental health in primary care. An epidemiological study of morbidity and use of health resources. Br J Psychiatry. 1997;170:529–35. 10.1192/bjp.170.6.529 9330019

[3] Parikh SV, Lin E, Lesage AD. Mental health treatment in Ontario: selected comparisons between the primary care and specialty sectors. Can J Psychiatry. 1997;42:929–34. 10.1177/070674379704200903 9429062

[4] Bodlund O, Andersson SO, Mallon L. Effects of consulting psychiatrist in primary care. 1-year follow-up of diagnosing and treating anxiety and depression. Scand J Prim Health Care. 1999;17:153–7. 10.1080/028134399750002566 10555244

[5] Nordstrom A, Bodlund O. Every third patient in primary care suffers from depression, anxiety or alcohol problems. Nord J Psychiatry. 2008;62:250–5. 10.1080/08039480802141129 18609025

[6] Hunsberger JG, Austin DR, Chen G, Manji HK. MicroRNAs in mental health: from biological underpinnings to potential therapies. Neuromolecular Med. 2009;11:173–82. 10.1007/s12017-009-8070-5 PMC2754593 19544012

[7] Mouillet-Richard S, Baudry A, Launay JM, Kellermann O. MicroRNAs and depression. Neurobiol Dis. 2012;46:272–8. 10.1016/j.nbd.2011.12.035 22226785

[8] Krishnan V, Nestler EJ. Linking molecules to mood: new insight into the biology of depression. Am J Psychiatry. 2010;167:1305–20. 10.1176/appi.ajp.2009.10030434 PMC3031089 20843874

[9] Coppen A. The biochemistry of affective disorders. Br J Psychiatry. 1967;113:1237–64. 10.1192/bjp.113.504.1237 4169954

[10] Lapin IP, Oxenkrug GF. Intensification of the central serotoninergic processes as a possible determinant of the thymoleptic effect. Lancet. 1969;1:132–6. 10.1016/s0140-6736(69)91140-4 4178247

[11] Lee S, Jeong J, Kwak Y, Park SK. Depression research: where are we now? Mol Brain. 2010;3:8. 10.1186/1756-6606-3-8 PMC2848031 20219105

[12] Ebmeier KP, Donaghey C, Steele JD. Recent developments and current controversies in depression. Lancet. 2006;367:153–67. 10.1016/S0140-6736(06)67964-6 16413879

[13] Chen X, Ba Y, Ma L, Cai X, Yin Y, et al. Characterization of microRNAs in serum: a novel class of biomarkers for diagnosis of cancer and other diseases. Cell Res. 2008;18:997–1006. 10.1038/cr.2008.282 18766170

[14] Mitchell PS, Parkin RK, Kroh EM, Fritz BR, Wyman SK, Pogosova-Agadjanyan EL, et al. Circulating microRNAs as stable blood-based markers for cancer detection. Proc Natl Acad Sci U S A. 2008;105:10513–8. 10.1073/pnas.0804549105 PMC2492472 18663219

[15] Pandey AK, Agarwal P, Kaur K, Datta M. MicroRNAs in diabetes: tiny players in big disease. Cell Physiol Biochem. 2009;23:221–32. 10.1159/000218169 19471090

[16] Ludwig N, Nourkami-Tutdibi N, Backes C, Lenhof HP, Graf N, Keller A, et al. 2015 Aug;62(8):1360-7. Circulating serum miRNAs as potential biomarkers for nephroblastoma. Pediatr Blood Cancer 10.1002/pbc.25481 25787821

[17] Zafari S, Backes C, Meese E, Keller A 2015 Feb 25 Circulating biomarker panels in Alzheimer’s disease. Gerontology 10.1159/000375236 25720553

[18] Ambros V. The functions of animal microRNAs. Nature. 2004;431:350–5. 10.1038/nature02871 15372042

[19] Miranda KC, Huynh T, Tay Y, Ang YS, Tam WL, Thomson AM, et al. A pattern-based method for the identification of microRNA binding sites and their corresponding heteroduplexes. Cell. 2006;126:1203–17. 10.1016/j.cell.2006.07.031 16990141

[20] Zeng Y. Regulation of the mammalian nervous system by microRNAs. Mol Pharmacol. 2009;75:259–64. 10.1124/mol.108.052118 PMC2684892 19004981

[21] Hebert SS, Horre K, Nicolai L, Papadopoulou AS, Mandemakers W, Silahtaroglu AN et al. Loss of microRNA cluster miR-29a/b-1 in sporadic Alzheimer’s disease correlates with increased BACE1/beta-secretase expression. Proc Natl Acad Sci U S A. 2008;105:6415–20. 10.1073/pnas.0710263105 PMC2359789 18434550

[22] Kim AH, Reimers M, Maher B, Williamson V, McMichael O, McClay JL et al. MicroRNA expression profiling in the prefrontal cortex of individuals affected with schizophrenia and bipolar disorders. Schizophr Res. 2010;124:183–91. 10.1016/j.schres.2010.07.002 PMC4373420 20675101

[23] Miller BH, Zeier Z, Xi L, Lanz TA, Deng S, Strathmann J, et al. MicroRNA-132 dysregulation in schizophrenia has implications for both neurodevelopment and adult brain function. Proc Natl Acad Sci U S A. 2012;109:3125–30. 10.1073/pnas.1113793109 PMC3286960 22315408

[24] Smalheiser NR, Lugli G, Rizavi HS, Torvik VI, Turecki G, Dwivedi Y. et al. MicroRNA expression is down-regulated and reorganized in prefrontal cortex of depressed suicide subjects. PLoS One. 2012;7:e33201. 10.1371/journal.pone.0033201 PMC3302855 22427989

[25] Weber JA, Baxter DH, Zhang S, Huang DY, Huang KH, Lee MJ et al. The microRNA spectrum in 12 body fluids. Clin Chem. 2010;56:1733–41. 10.1373/clinchem.2010.147405 PMC4846276 20847327

[26] Gibbings DJ, Ciaudo C, Erhardt M, Voinnet O. Multivesicular bodies associate with components of miRNA effector complexes and modulate miRNA activity. Nat Cell Biol. 2009;11:1143–9. 10.1038/ncb1929 19684575

[27] Arroyo JD, Chevillet JR, Kroh EM, Ruf IK, Pritchard CC, Gibson DF et al. Argonaute2 complexes carry a population of circulating microRNAs independent of vesicles in human plasma. Proc Natl Acad Sci U S A. 2011;108:5003–8. 10.1073/pnas.1019055108 PMC3064324 21383194

[28] Vickers KC, Palmisano BT, Shoucri BM, Shamburek RD, Remaley AT. MicroRNAs are transported in plasma and delivered to recipient cells by high-density lipoproteins. Nat Cell Biol. 2011;13:423–33. 10.1038/ncb2210 PMC3074610 21423178

[29] Valadi H, Ekstrom K, Bossios A, Sjostrand M, Lee JJ, Lötvall JO et al. Exosome-mediated transfer of mRNAs and microRNAs is a novel mechanism of genetic exchange between cells. Nat Cell Biol. 2007;9:654–9. 10.1038/ncb1596 17486113

[30] Wang X, Sundquist J, Zoller B, Memon AA, Palmer K, Sundquist K et al. Determination of 14 circulating microRNAs in Swedes and Iraqis with and without diabetes mellitus type 2. PLoS One. 2014;9, e86792. 10.1371/journal.pone.0086792 PMC3907562 24497980

[31] Belzeaux R, Bergon A, Jeanjean V, Loriod B, Formisano-Treziny C, Verrier L et al. Responder and nonresponder patients exhibit different peripheral transcriptional signatures during major depressive episode. Transl Psychiatry. 2012;2, e185. 10.1038/tp.2012.112 PMC3565773 23149449

[32] Rong H, Liu TB, Yang KJ, Yang HC, Wu DH, Liao CP et al. MicroRNA-134 plasma levels before and after treatment for bipolar mania. J Psychiatr Res. 2011;45:92–5. 10.1016/j.jpsychires.2010.04.028 20546789

[33] Bocchio-Chiavetto L, Maffioletti E, Bettinsoli P, Giovannini C, Bignotti S, Tardito D et al. Blood microRNA changes in depressed patients during antidepressant treatment. Eur Neuropsychopharmacol. 2013;23:602–11. 10.1016/j.euroneuro.2012.06.013 22925464

[34] Sundquist J, Lilja A, Palmer K, Memon AA, Wang X, Johansson LM et al. 2015 Feb;206(2):128-35 Mindfulness group therapy in primary care patients with depression, anxiety and stress and adjustment disorders: randomised controlled trial. Br J Psychiatry 10.1192/bjp.bp.114.150243 25431430

[35] Eller T, Vasar V, Shlik J, Maron E. Pro-inflammatory cytokines and treatment response to escitalopram in major depressive disorder. Prog Neuropsychopharmacol Biol Psychiatry. 2008;32:445–50. 10.1016/j.pnpbp.2007.09.015 17976882

[36] Croft HA, Pomara N, Gommoll C, Chen D, Nunez R, Mathews M et al. Efficacy and safety of vilazodone in major depressive disorder: a randomized, double-blind, placebo-controlled trial. J Clin Psychiatry. 2014;75:e1291–1298. 10.4088/JCP.14m08992 25470094

[37] Svanborg P, Asberg M. A comparison between the Beck Depression Inventory (BDI) and the self-rating version of the Montgomery Asberg Depression Rating Scale (MADRS). J Affect Disord. 2001;64:203–16. 10.1016/s0165-0327(00)00242-1 11313087

[38] Callahan EJ, Bertakis KD, Azari R, Helms LJ, Robbins J, Miller J et al. Depression in primary care: patient factors that influence recognition. Fam Med. 1997;29:172–6. 9085097

[39] Kerr LK, Kerr Jr LD. Screening tools for depression in primary care: the effects of culture, gender, and somatic symptoms on the detection of depression. West J Med. 2001;175:349–52. 10.1136/ewjm.175.5.349 PMC1071624 11694495

[40] Schneider B, Prvulovic D. Novel biomarkers in major depression. Curr Opin Psychiatry. 2013;26:47–53. 10.1097/YCO.0b013e32835a5947 23154643

[41] Kosaka N, Iguchi H, Yoshioka Y, Takeshita F, Matsuki Y, Ochiya T et al. Secretory mechanisms and intercellular transfer of microRNAs in living cells. J Biol Chem. 2010;285:17442–52. 10.1074/jbc.M110.107821 PMC2878508 20353945

[42] Laterza OF, Lim L, Garrett-Engele PW, Vlasakova K, Muniappa N, Tanaka WK et al. Plasma MicroRNAs as sensitive and specific biomarkers of tissue injury. Clin Chem. 2009;55:1977–83. 10.1373/clinchem.2009.131797 19745058

[43] Landgraf P, Rusu M, Sheridan R, Sewer A, Iovino N, Aravin A et al. A mammalian microRNA expression atlas based on small RNA library sequencing. Cell. 2007;129:1401–14. 10.1016/j.cell.2007.04.040 PMC2681231 17604727

[44] Lim LP, Lau NC, Garrett-Engele P, Grimson A, Schelter JM, Castle J et al. Microarray analysis shows that some microRNAs downregulate large numbers of target mRNAs. Nature. 2005;433:769–73. 10.1038/nature03315 15685193

[45] Zhou R, Yuan P, Wang Y, Hunsberger JG, Elkahloun A, Wei Y et al. Evidence for selective microRNAs and their effectors as common long-term targets for the actions of mood stabilizers. Neuropsychopharmacology. 2009;34:1395–405. 10.1038/npp.2008.131 PMC2669666 18704095

[46] Katsuura S, Kuwano Y, Yamagishi N, Kurokawa K, Kajita K, Akaike Y et al. MicroRNAs miR-144/144* and miR-16 in peripheral blood are potential biomarkers for naturalistic stress in healthy Japanese medical students. Neurosci Lett. 2012;516:79–84. 10.1016/j.neulet.2012.03.062 22484483

[47] Honda M, Kuwano Y, Katsuura-Kamano S, Kamezaki Y, Fujita K, Akaike Y et al. Chronic academic stress increases a group of microRNAs in peripheral blood. PLoS One. 2013;8:e75960. 10.1371/journal.pone.0075960 PMC3794012 24130753

[48] Persengiev S, Kondova I, Otting N, Koeppen AH, Bontrop RE. Genome-wide analysis of miRNA expression reveals a potential role for miR-144 in brain aging and spinocerebellar ataxia pathogenesis. Neurobiol Aging. 2011;32(2316):e2317–2327. 10.1016/j.neurobiolaging.2010.03.014 20451302

[49] Machado-Vieira R, Salvadore G, DiazGranados N, Ibrahim L, Latov D, Wheeler-Castillo C et al. New therapeutic targets for mood disorders. Sci World J. 2010;10:713–26. 10.1100/tsw.2010.65 PMC3035047 20419280

[50] Dwivedi Y. Emerging role of microRNAs in major depressive disorder: diagnosis and therapeutic implications. Dialogues Clin Neurosci. 2014;16:43–61. 10.31887/DCNS.2014.16.1/ydwivedi PMC3984890 24733970

[51] Keller A, Leidinger P, Vogel B, Backes C, ElSharawy A, Galata V et al. miRNAs can be generally associated with human pathologies as exemplified for miR-144*. BMC Med. 2014;12:224. 10.1186/s12916-014-0224-0 PMC4268797 25465851

[52] Leidinger P, Backes C, Deutscher S, Schmitt K, Mueller SC, Frese K et al. A blood based 12-miRNA signature of Alzheimer disease patients. Genome Biol. 2013;14:R78. 10.1186/gb-2013-14-7-r78 PMC4053778 23895045

[53] Oei TP, Bullbeck K, Campbell JM. Cognitive change process during group cognitive behaviour therapy for depression. J Affect Disord. 2006;92:231–41. 10.1016/j.jad.2006.02.004 16542734

[54] Eller T, Vasar V, Shlik J, Maron E. Effects of bupropion augmentation on pro-inflammatory cytokines in escitalopram-resistant patients with major depressive disorder. J Psychopharmacol. 2009;23:854–8. 10.1177/0269881108091077 18562403

[55] Mestdagh P, Van Vlierberghe P, De Weer A, Muth D, Westermann F, Speleman F et al. A novel and universal method for microRNA RT-qPCR data normalization. Genome Biol. 2009;10:R64. 10.1186/gb-2009-10-6-r64 PMC2718498 19531210

[56] Chang TC, Yu D, Lee YS, Wentzel EA, Arking DE, West KM et al. Widespread microRNA repression by Myc contributes to tumorigenesis. Nat Genet. 2008;40:43–50. 10.1038/ng.2007.30 PMC2628762 18066065

[57] Pradervand S, Weber J, Thomas J, Bueno M, Wirapati P, Lefort K et al. Impact of normalization on miRNA microarray expression profiling. RNA. 2009;15:493–501. 10.1261/rna.1295509 PMC2657010 19176604

[58] Blondal T, Jensby Nielsen S, Baker A, Andreasen D, Mouritzen P, Wrang Teilum M et al. Assessing sample and miRNA profile quality in serum and plasma or other biofluids. Methods. 2013;59:S1–6. 10.1016/j.ymeth.2012.09.015 23036329

[59] Song J, Bai Z, Han W, Zhang J, Meng H, Bi J et al. Identification of suitable reference genes for qPCR analysis of serum microRNA in gastric cancer patients. Dig Dis Sci. 2012;57:897–904. 10.1007/s10620-011-1981-7 22198701

