# Supplementary material for: Circulating microRNA-144-5p is associated with depressive disorders
Source: Clin Epigenetics. 2015 Jul 22;7(1):69. doi: 10.1186/s13148-015-0099-8 (PMC4509564; doi:10.1186/s13148-015-0099-8)
Supplement: Additional file 1: — Supplementary Tables. Table S1. six candidate miRNAs with >1.5 fold differential expression between baseline and follow-up and changes in more than half of the samples (n≥6) were selected for further validation. Table S2a and Table S2b. The relationships between miR-144-5p and two depression scores at baseline, HADS-D (β=-0.02, p=0.02) and PHQ-9 (β=-0.02, p=0.06) were also significant or borderline significant, whereas the association with HADS-A score (β=-0.01, p=0.37) was non-significant (Table S2c). [file 13148_2015_99_MOESM1_ESM.doc]

**Supplementary Tables and Figure**

Supplementary Table S1: Six candidate miRNAs with >1.5 fold differential expression between baseline and follow-up and changes in more than half of the samples (n≥6) were selected for further validation.

Supplementary Table S2a and Table S2b: The relationships between miR-144-5p and two depression scores at baseline, HADS-D (β=-0.02, p=0.02) and PHQ-9 (β=-0.02, p=0.06) were also significant or borderline significant, whereas the association with HADS-A score (β=-0.01, p=0.37) was non-significant (Table S2c).

Supplementary Figure S1: The selected 5 miRNAs were validated in all the 169 patients at baseline and follow up. After treatment, the mean plasma miR-144-5p level and miR-30a-5p in the depression/anxiety patients increased significantly compared to baseline, (p<0.0001 and p=0.007, respectively).

| Table S1. Statistical test of selected miRNAs | | |  |
| --- | --- | --- | --- |
| ***miRNA*** | ***p-value**** | ***Median FC***** | ***Nr ****** |
| hsa-miR-144-5p | 0.09 | 1.65 | 7 |
| hsa-miR-885-5p | 0.08 | 1.60 | 6 |
| hsa-miR-92b-3p | 0.21 | 1.60 | 6 |
| hsa-miR-29b-2-5p | 0.37 | 0.58 | 7 |
| hsa-miR-29a-5p | 0.04 | 0.58 | 6 |
| hsa-miR-30a-5p | 0.04 | 0.65 | 6 |
| Reference gene = global mean of all miRNAs | | | |
| *Wilcoxon signed rank test between baseline and after treatment | | | |
| **FC = 2-∆∆Ct |  |  |  |
| ***Number of samples with FC >= 1.5 or <=0.67   | **Table S2a.** Associations between selected miRNAs (∆Ct at baseline) and HADS-D for the whole cohort, as determined using linear regression | | | | | | | | --- | --- | --- | --- | --- | --- | --- | |  | **Univariate analysis** | | | **Adjusted analysis** | | | |  | **β** | **p-value** | **95% CI** | **β** | **p-value** | **95% CI** | | *miR-144-5p* (n=169) |  |  |  |  |  |  | | HADS-D at baseline | -0.03 | 0.046 | -0.06; -0.0006 | -0.03 | 0.02 | -0.06; -0.006 | | Sex (female vs. male) |  |  |  | -0.37 | 0.02 | -0.67; -0.06 | | Age |  |  |  | 0.007 | 0.14 | -0.002; 0.02 | | *miR-29a-5p* (n=162)^a^ |  |  |  |  |  |  | | HADS-D at baseline | 0.02 | 0.13 | -0.007; 0.05 | 0.03 | 0.07 | -0.02; 0.06 | | Sex (female vs. male) |  |  |  | 0.02 | 0.92 | -0.33; 0.37 | | Age |  |  |  | -0.01 | 0.02 | -0.02; -0.002 | | *miR-29b-2-5p* (n=162)^a^ |  |  |  |  |  |  | | HADS-D at baseline | 0.007 | 0.66 | -0.02; 0.04 | 0.005 | 0.75 | -0.02; 0.03 | | Sex (female vs. male) |  |  |  | -0.17 | 0.35 | -0.52; 0.18 | | Age |  |  |  | 0.005 | 0.37 | -0.006; 0.01 | | *miR-30a-5p* (n=162)^a^ |  |  |  |  |  |  | | HADS-D at baseline | 0.01 | 0.31 | -0.01; 0.04 | 0.01 | 0.26 | -0.01; 0.04 | | Sex (female vs. male) |  |  |  | 0.26 | 0.10 | -0.05; 0.57 | | Age |  |  |  | -0.0001 | 0.98 | -0.009; 0.009 | | *miR-92b-3p* (n=169) |  |  |  |  |  |  | | HADS-D at baseline | 0.005 | 0.69 | -0.02; 0.03 | 0.008 | 0.52 | -0.02; 0.03 | | Sex (female vs. male) |  |  |  | 0.05 | 0.74 | -0.22; 0.32 | | Age |  |  |  | -0.004 | 0.24 | -0.01; 0.004 |   ^a^Information (clinical or qRT-PCR) is missing for some patients (Table S2a,b,c)   | **Table S2b.** Associations between selected miRNAs (∆Ct at baseline) and HADS-A for the whole cohort, as determined using linear regression | | | | | | | | --- | --- | --- | --- | --- | --- | --- | |  | **Univariate analysis** | | | **Adjusted analysis** | | | |  | **β** | **p-value** | **95% CI** | **β** | **p-value** | **95% CI** | | *miR-144-5p* (n=169) |  |  |  |  |  |  | | HADS-A at baseline | -0.01 | 0.39 | -0.04; 0.02 | -0.01 | 0.37 | -0.04; 0.02 | | Sex (female vs. male) |  |  |  | -0.34 | 0.03 | -0.65; -0.04 | | Age |  |  |  | 0.005 | 0.32 | -0.005; 0.01 | | *miR-29a-5p* (n=162)^a^ |  |  |  |  |  |  | | HADS-A at baseline | 0.03 | 0.05 | -0.0004; 0.06 | 0.03 | 0.08 | -0.004; 0.06 | | Sex (female vs. male) |  |  |  | -0.01 | 0.95 | -0.36; 0.34 | | Age |  |  |  | -0.009 | 0.08 | -0.02; 0.001 | | *miR-29b-2-5p* (n=162)^a^ |  |  |  |  |  |  | | HADS-A at baseline | 0.02 | 0.24 | -0.01; 0.05 | 0.03 | 0.11 | -0.006; 0.06 | | Sex (female vs. male) |  |  |  | -0.17 | 0.34 | -0.51; 0.18 | | Age |  |  |  | 0.007 | 0.19 | -0.004; 0.02 | | *miR-30a-5p* (n=162)^a^ |  |  |  |  |  |  | | HADS-A at baseline | 0.01 | 0.35 | -0.01; 0.04 | 0.01 | 0.37 | -0.02; 0.04 | | Sex (female vs. male) |  |  |  | 0.24 | 0.12 | -0.06; 0.55 | | Age |  |  |  | 0.001 | 0.77 | -0.008; 0.01 | | *miR-92b-3p* (n=169) |  |  |  |  |  |  | | HADS-A at baseline | 0.001 | 0.92 | -0.02; 0.03 | 0.003 | 0.84 | -0.02; 0.03 | | Sex (female vs. male) |  |  |  | 0.04 | 0.77 | -0.23; 0.31 | | Age |  |  |  | -0.004 | 0.41 | -0.01; 0.005 |  | **Table S2c.** Associations between selected miRNAs (∆Ct at baseline) and PHQ-9 for the whole cohort, as determined using linear regression | | | | | | | | --- | --- | --- | --- | --- | --- | --- | |  | **Univariate analysis** | | | **Adjusted analysis** | | | |  | **β** | **p-value** | **95% CI** | **β** | **p-value** | **95% CI** | | *miR-144-5p* (n=169) |  |  |  |  |  |  | | PHQ-9 at baseline | -0.02 | 0.06 | -0.03; 0.0009 | -0.02 | 0.06 | -0.03; 0.001 | | Sex (female vs. male) |  |  |  | -0.31 | 0.04 | -0.62; -0.01 | | Age |  |  |  | 0.006 | 0.24 | -0.004; 0.01 | | *miR-29a-5p* (n=162)^a^ |  |  |  |  |  |  | | PHQ-9 at baseline | 0.01 | 0.21 | -0.007; 0.03 | 0.01 | 0.16 | -0.006; 0.03 | | Sex (female vs. male) |  |  |  | -0.03 | 0.86 | -0.38; 0.32 | | Age |  |  |  | -0.01 | 0.03 | -0.02; -0.0009 | | *miR-29b-2-5p* (n=162)^a^ |  |  |  |  |  |  | | PHQ-9 at baseline | -0.008 | 0.42 | -0.03; 0.01 | -0.006 | 0.56 | -0.03; 0.01 | | Sex (female vs. male) |  |  |  | -0.17 | 0.35 | -0.51; 0.18 | | Age |  |  |  | 0.005 | 0.38 | -0.006; 0.01 | | *miR-30a-5p* (n=162)^a^ |  |  |  |  |  |  | | PHQ-9 at baseline | 0.004 | 0.63 | -0.01; 0.02 | 0.002 | 0.79 | -0.01; 0.02 | | Sex (female vs. male) |  |  |  | 0.24 | 0.13 | -0.07; 0.55 | | Age |  |  |  | 0.0004 | 0.93 | -0.009; 0.009 | | *miR-92b-3p* (n=169) |  |  |  |  |  |  | | PHQ-9 at baseline | -0.006 | 0.49 | -0.02; 0.01 | -0.004 | 0.63 | -0.02; 0.01 | | Sex (female vs. male) |  |  |  | 0.04 | 0.75 | -0.23; 0.31 | | Age |  |  |  | -0.004 | 0.36 | -0.01; 0.004 | | | | |
