# Supplementary material for: Circulating microRNA-144-5p is associated with depressive disorders
Source: Clin Epigenetics. 2015 Jul 22;7(1):69. doi: 10.1186/s13148-015-0099-8 (PMC4509564; doi:10.1186/s13148-015-0099-8)
Supplement: Additional file 2: Figure S1. — Supplementary Figure. Plasma 5 miRNAs levels (determined by the 2ΔCt method)changed after treatment. Data are shown as the mean and 95 % CI. P calculated with paired t-test [file 13148_2015_99_MOESM2_ESM.pdf]

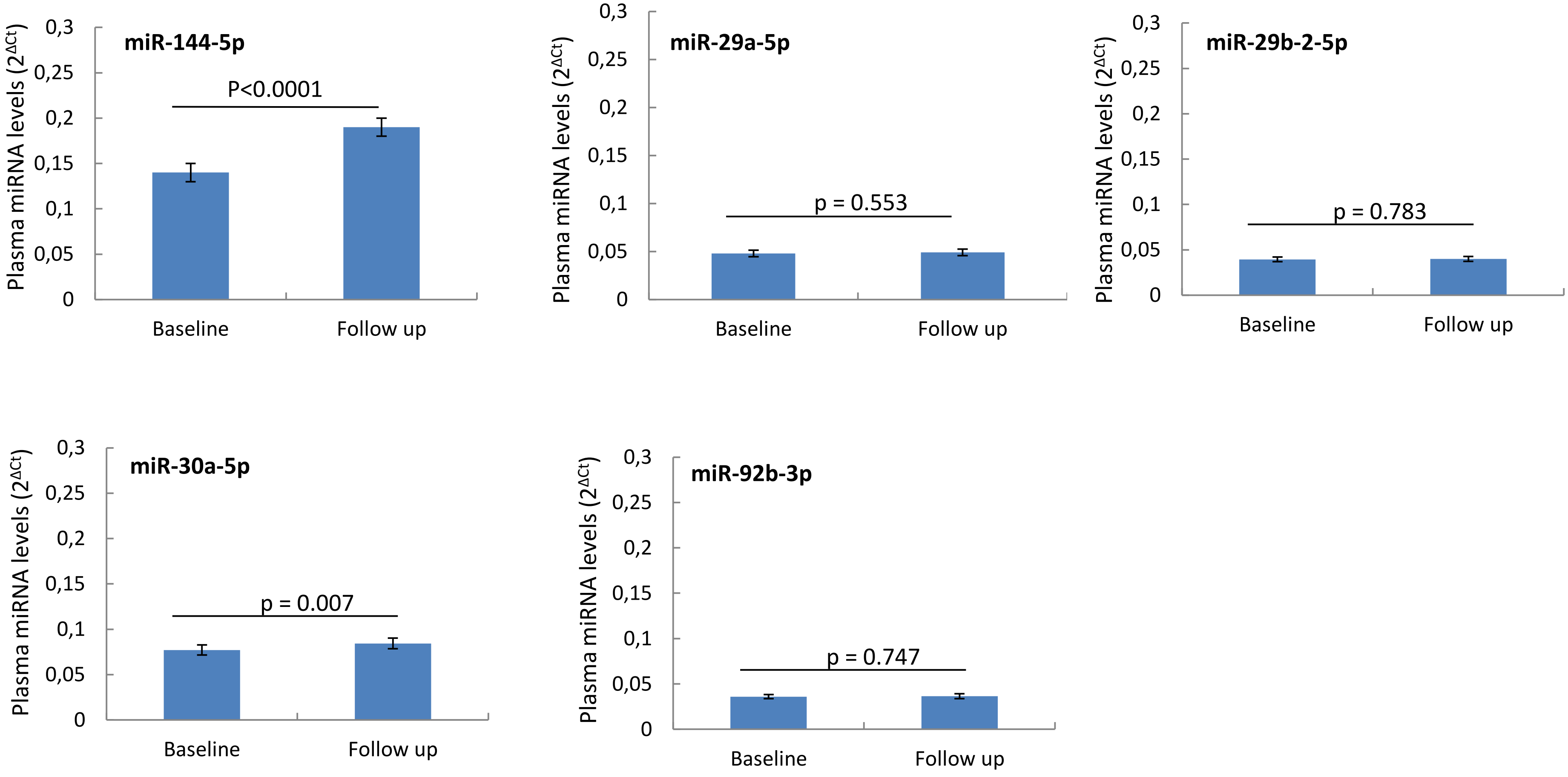

Figure S1. Plasma 5 miRNAs levels (determined by the 2ΔCt method)changed after treatment. Data are shown as the mean and 95% CI. P calculated with paired t-test.
